# Pteridophyte species richness in the central Himalaya is limited by cold climate extremes at high elevations and rainfall seasonality at low elevations

**DOI:** 10.1002/ece3.8958

**Published:** 2022-05-24

**Authors:** Hong Qian, Michael Kessler, Ole R. Vetaas

**Affiliations:** ^1^ 11057 Research and Collections Center Illinois State Museum Springfield Illinois USA; ^2^ 27217 Department of Systematic Botany University of Zurich Zurich Switzerland; ^3^ Department of Geography University of Bergen Bergen Norway

**Keywords:** climatic condition, environmental gradient, fern, lycophyte, seasonality, species diversity, stressful climate

## Abstract

There is a consensus that climate factors strongly influence species richness along elevation gradients, but which factors are crucial and how they operate are still elusive. Here, we assess the relative importance of temperature‐related versus precipitation‐related variables and the relative importance of extreme climate versus climate seasonality in driving pteridophyte species richness. We used correlation and regression analyses to relate species richness of pteridophytes, and their two major groups (lycophytes, ferns), in fifty 100‐m vertical bands to climatic factors representing different aspects of climatic conditions (general climate, stressful climate, and climate seasonality). Variation partitioning analysis was used to determine the relative importance of each group of climatic factors on species richness. Across the entire elevational gradient, species richness had a parabolic response to mean annual temperature (adjusted *R* ^2^ = .87−.91), and a linear response to annual precipitation (adjusted *R* ^2^ = .82). Mean annual temperature and annual precipitation in the second‐order polynomial model together explained 96.3%−98.7% of the variation in species richness. The variation in species richness uniquely explained by minimum temperature of the coldest month was much greater than that uniquely explained by temperature seasonality, but the variation in species richness uniquely explained by precipitation during the driest month was much smaller than that uniquely explained by precipitation seasonality. Overall, extreme climate variables explained slightly more variation than did climate seasonality. Our study suggests that pteridophyte richness along the elevational gradient is largely driven by a combination of both temperature‐ and precipitation‐related parameters, although precipitation‐related variables play a slightly stronger role, and that extreme low temperature events (at high elevations) and seasonal precipitation variability (at low elevations) are the strongest determinants of pteridophyte species richness.

## Graphical abstract

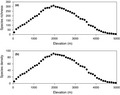

## 1 INTRODUCTION

One of the central aims in ecology, biogeography, and biodiversity conservation is to understand the mechanisms that drive the variation in species diversity among areas. Species diversity generally varies between areas with different climatic conditions (Rosenzweig, 1995), and commonly decreases with decreasing temperature and precipitation (Rosenzweig, 1995), which are widely considered as major drivers of species diversity (Currie et al., 2004). A well‐known pattern of species diversity is that species richness decreases with increasing latitude (i.e., the latitudinal diversity gradient; Rosenzweig, 1995). Because this pattern is consistent with patterns of some climatic factors, it is thought that the latitudinal diversity gradient is largely driven by climatic factors (Currie et al., 2004). Yet, the latitudinal pattern also has some deviations. In particular, at 30° north and south, there are deserts and semiarid regions around the globe. As a result, in Africa, species richness decreases from the equatorial wet tropics toward the desert zones, although mean and extreme temperatures increase (Kreft & Jetz, 2007). This demonstrates two crucial facts, namely that precipitation is a vital resource gradient and that temperature in contrast is a regulator gradient (Vetaas, 2021). Thermal energy regulates liquid water availability by evapotranspiration where both high and low values will transform the liquid water into either gas or solid ice, respectively (O'Brien, 1993, 2006).

Elevational diversity gradients are as ubiquitous as latitudinal diversity gradients (Sanders & Rahbek, 2012). Elevational gradients have been considered better systems, compared to latitudinal gradients, for testing hypotheses on the relationships between species diversity and climatic conditions for multiple reasons (Qian et al., 2020). Most importantly, many of the potential underlying causes that covary along latitudinal gradients (e.g., history, climate, time since glaciation) do not covary when one considers several elevational gradients in combination (Körner, 2007; Sanders & Rahbek, 2012). Further, geographical distances are much shorter along elevational gradients, so that dispersal limitation and geographical barriers play a lesser role, implying that species distributions along an elevational gradient are expected to reflect their climatic niches more directly than along a latitudinal gradient (Qian et al., 2020; Vetaas, 2021). Different elevational gradients in many mountain systems across the world may therefore be considered as replicates in hypothesis testing (Vetaas, 2021). Because many of the biodiversity hotspots across the world are located in montane regions (Myers et al., 2000), understanding the underlying mechanisms driving species diversity patterns along elevational gradients is of major interest in ecology and biogeography (Fjeldså et al., 2012; Rahbek et al., 2019).

Unlike most of the latitudinal diversity gradients which show monotonic relationships between species diversity and latitude, most elevational diversity gradients, particularly those located in tropical and subtropical latitudes, show hump‐shaped elevational diversity patterns (Rahbek, 1995, 2005), that is, the elevations with the highest species diversity are located at some intermediate elevation of the gradient. The causes of the hump‐shaped patterns of species diversity are not yet fully understood; they include a number of potential factors, which may be roughly grouped into spatial, evolutionary/historical, and climatic factors.

Among spatial factors, for example, species richness may be highest at the middle of an elevational gradient because species from both low and high elevation assemblages disperse into the middle of the gradient and overlap there, creating a richness peak (Kessler et al., 2011). A somewhat related idea proposes that along geometrically bounded gradients, as presented by elevational gradients between sea level and mountain tops, species with extensive elevational range amplitudes will be forced to overlap in the middle, creating a mid‐elevation richness hump (Colwell et al., 2016; Colwell & Lees, 2000). Also, elevational richness patterns are influenced by land surface area, although this is unlikely to create a mid‐elevation richness hump (Karger et al., 2011; Romdal & Grytnes, 2007).

Evolutionary and historical factors consider such aspects as the time lag of taxa to adapt to novel environmental conditions as are offered when new mountain systems are formed. Thus, the richness pattern in a mountain range may be influenced by the age of the mountain range in combination with the speed at which taxa can diversify (Quintero & Jetz, 2018). At a long temporal scale, the mid‐elevation zone will always be far away from areas that may be affected by water scarcity due to overheating (desertification) or by glaciations, reducing extinction risk (Vetaas et al., 2019). All of these factors may in turn influence the phylogenetic composition of mountain assemblages (Hernández‐Rojas et al., 2021).

The most commonly considered factors for explaining mountain richness patterns, however, are related to climate and its influence on both the distribution of individual species and ecosystem properties such as productivity (Kessler et al., 2014). For instance, because precipitation is highest somewhere between low and high elevations in many mountain systems, causing a hump‐shaped elevational pattern in precipitation that mirrors the common diversity pattern, water availability has been considered to be a major driver of the variation of species diversity along elevational gradients (Bhattarai et al., 2004; Kessler, Kluge, et al., 2011; Kluge et al., 2006). It is likely that the interplay of temperature and precipitation is a major driver of species diversity along most elevational gradients (Bhatta et al., 2021), but their relative importance might vary not only between different mountain systems but also between different elevational segments within a single mountain system. On the one hand, species distributional limits at high latitudes and elevations are often driven by their tolerance to low temperatures (Körner, 2021; Ricklefs, 2001). Because fewer species can tolerate relatively low temperatures, species diversity is in general lower in areas with such temperatures (“cold tolerance hypothesis”; Farrell et al., 1992; Wang et al., 2011). On the other hand, temperature seasonality has also been considered a major climatic factor determining the northern limit of the latitudinal range of a species in the Northern Hemisphere, which has been formulated as the “temperature seasonality tolerance hypothesis” or, more generally, the “climate variability hypothesis” (Stevens, 1989). For example, Wu et al. (2014) found that temperature seasonality is the strongest explanatory factor for babbler species richness along an elevational gradient in China; similarly, Wiens et al. (2006) showed that temperature seasonality, rather than low temperatures, constrains species range limits at higher latitudes for hylid frogs in North America. However, few studies have tested whether climate stress is a stronger driver of species diversity than climate seasonality, and vice versa. Because the correlation between temperature (including minimum temperature of the coldest month) and temperature seasonality is much weaker along an elevational gradient, particularly in a tropical or subtropical region, than that along a latitudinal gradient (Janzen, 1967), elevational gradients appear to be ideal systems for testing the relative importance of stressful climates and climate seasonality in driving species diversity.

The Himalaya is a global biodiversity hotspot, and the elevational gradient of the central Himalaya within Nepal, which spans over 8700 m, is the longest elevational gradient in the world. It includes a full series of life zones from tropical forests at low elevations to nival areas where the temperature is too low for vascular plant survival. Accordingly, it is one of the best elevational gradients for testing ecological and biogeographical hypotheses on species distributions across climate gradients. For this reason, several previous studies have investigated the relationships between taxonomic and phylogenetic diversity of plants and climate in relation to elevation in the central Himalaya (e.g., Bhattarai & Vetaas, 2003; Bhattarai et al., 2004; Grytnes & Vetaas, 2002; Qian et al., 2019; Rana et al., 2019).

Pteridophytes, including both lycophytes and ferns, are considered to be good climate indicators (Khine et al., 2019) because they are dispersed by spores which can travel thousands of kilometers by wind (Wolf et al., 2001), so that their distributions are more in equilibrium with climate than distributions of seed plants (Qian, 2009), and because the stomatal control of pteridophytes is less efficient than that of angiosperms (Brodribb & McAdam, 2011), so that they appear to be more susceptible to limited water availability (Hernández‐Rojas et al., 2020). Across latitudinal gradients, species richness of pteridophytes generally decreases polewards (Karger et al., 2011), whereas on elevational gradients, species richness of pteridophytes commonly peaks at mid‐elevations (Bhattarai et al., 2004; Khine et al., 2019; Tanaka & Sato, 2013; Tang et al., 2014). These patterns have been interpreted as being driven by climatic factors (Kessler, Kluge, et al., 2011; Khine et al., 2019; Kluge et al., 2006). In general, species richness of pteridophytes is highest in cool and humid habitats and is low not only in cold and arid habitats but also in hot habitats, because high temperatures lead to water stress even in areas of high precipitation (Kessler et al., 2011; Kessler, Kluge, et al., 2011; Khine et al., 2019; Kluge et al., 2006). Accordingly, while it is commonly found that temperature is more strongly correlated with species richness of angiosperms than precipitation (Moles et al., 2014), several studies have found the opposite for pteridophytes (Bickford & Laffan, 2006; Kessler, 2001; Kessler, Kluge, et al., 2011; Kreft et al., 2010; Qian et al., 2021). However, few studies have assessed the relative importance of these two climatic factors in driving species richness of pteridophytes, and further studies are needed to assess whether precipitation is indeed a more important driver of species richness of pteridophytes, compared to temperature. Because temperature is often weakly correlated with precipitation across long elevational gradients (Qian, Sandel, et al., 2019), long elevational gradients appear to be ideal systems for testing the relative importance of these two climatic factors on species richness.

In this study, we determine the relative importance of temperature‐related versus precipitation‐related variables and the relative importance of extreme climate versus climate seasonality in driving pteridophyte species richness in the central Himalaya. Specifically, we address two questions. First, are precipitation‐related climatic variables more important drivers of pteridophyte species richness than temperature‐related climatic variables? We predict that precipitation‐related variables play more important roles than temperature‐related variables in shaping patterns of pteridophyte species richness along the elevational gradient of the central Himalaya (H1). Second, are climate extreme variables (e.g., minimum temperature in winter, minimum precipitation in the driest season) more important drivers of pteridophyte species richness than are climate seasonality variables (e.g., temperature seasonality, precipitation seasonality)? Considering that previous studies have shown that minimum temperature is more strongly related to distributions and richness of seed plants, compared with temperature seasonality (e.g., Wang et al., 2011), we predict that climate extreme variables play more important roles than climate seasonality variables in shaping patterns of pteridophyte species richness along the elevational gradient of the central Himalaya (H2).

Bhattarai et al. (2004) have studied the relationships between pteridophyte species richness and climate in Nepal. However, their analysis, which was based on the best data available at that time (Iwatsuki, 1988), included only about half of the pteridophyte species known today to occur in Nepal (293 species in their study versus 534 species in our study), and their study did not address the questions posed here, namely the relative importance of temperature‐related versus precipitation‐related variables and the relative importance of extreme climate versus climate seasonality in driving pteridophyte species richness. Thus, our study is an important extension of their study and closes a critical knowledge gap. We expect that the relationships between species richness and various climatic factors reported in this study are important information for developing biodiversity conservation plans.

## 2 MATERIALS AND METHODS

### 2.1 Study area and plant data

The elevational gradient of the central Himalaya defined in this study covers the whole of Nepal, which is located at 80°04′–88°12′E and 26°22′–30°27′N, with an elevational gradient ranging from 60–8848 m.a.s.l. We obtained the species list and elevational ranges of pteridophytes in Nepal from “Ferns and Fern‐allies of Nepal” (Fraser‐Jenkins & Kandel, 2019; Fraser‐Jenkins et al., 2015; Kandel & Fraser‐Jenkins, 2020). Data for elevational ranges of pteridophytes within Nepal were supplemented with the data published in Iwatsuki (1988), Bista et al. (2002), and Gurung (1991, 1998). We standardized the botanical nomenclature of the Nepalese pteridophytes according to World Ferns (https://www.worldplants.de/; Hassler, 2004−2021). Infraspecific taxa were combined at the species level. Non‐native species were excluded. The final data set included 534 species of pteridophytes, of which 38 belong to the class Lycopodiopsida (commonly known as lycophytes) and 496 to the class Polypodiopsida (commonly known as ferns). These species belong to 117 genera and 34 families (Table S1).

Pteridophyte species are distributed from 60 m to around 5000 m in elevation in Nepal and we divided the elevation gradient into fifty 100‐m vertical bands with the lowest band being located at 0–100 m above sea level. Following previous studies (e.g., Vetaas & Grytnes, 2002), a species was assigned to all vertical bands which were entirely or partially located within the known elevational range of the species. Lycophytes were distributed in 45 bands and fern were distributed in all the 50 bands.

### 2.2 Climate data

Mean annual temperature and annual precipitation are commonly considered as determinants of species richness at broad spatial extents (Kooyman et al., 2012), including pteridophytes along elevational gradients (e.g., Tanaka & Sato, 2014). Minimum temperature of the coldest month and precipitation during the driest month, which represent, or are strongly correlated to, extreme and stressful climates, and temperature seasonality and precipitation seasonality, which represent intra‐annual variability of climate, also constrain the distributions of species (Weigelt et al., 2015). These six climatic variables have been commonly considered as the most important climatic factors determining distributions and diversity of plants, including pteridophytes (Hernández‐Rojas et al., 2020; Khine et al., 2019; Qian et al., 2021; Salazar et al., 2015). We obtained climate data from CHELSA (https://chelsa‐climate.org/bioclim; Karger et al., 2017) for bio1, bio4, bio6, bio12, bio14, and bio15, which correspond, respectively, to mean annual temperature, temperature seasonality, minimum temperature of the coldest month, annual precipitation, precipitation during the driest month, and precipitation seasonality. We considered bio1, bio6, and bio4 as a set of temperature‐related variables, and bio12, bio14 and bio15 as a set of precipitation‐related variables. We further considered bio6 and 14 as extreme climatic variables and bio4 and bio15 as climatic seasonality variables. The mean value of each of the six variables was calculated for each elevational band using 30‐arc‐second resolution data.

### 2.3 Data analysis

All variables were standardized to have mean = 0 and SD = 1 before statistical analyses. We conducted simple and multiple regression analyses, both with first‐ and second‐order polynomials, to assess the relationships between species richness and climatic variables, based on the adjusted coefficient of determination for each regression model and standardized regression coefficients for explanatory variables in each multiple regression model. To determine whether temperature‐related variables have a stronger effect on species richness than do precipitation‐related variables and vice versa, we conducted a series of partial regressions (Legendre & Legendre, 2012) to partition the explained variation into three portions: explained uniquely by temperature‐related variables, explained uniquely by precipitation‐related variables, and explained jointly by the temperature‐ and precipitation‐related variables. To determine independent and shared effects of extreme climate (bio6, bio14) and climate variability (bio4, bio15) on species richness, we conducted a second set of partial regressions based on the same approach. Furthermore, to determine independent and shared effects of the general climate (bio1, bio12), extreme climate (bio6, bio14), and climate variability (bio4, bio15) on species richness, we conducted a third set of partial regressions to partition the variance in species richness explained by each of the three types of climatic variables independently and by two or three types of climatic variables jointly.

To find the best climate model explaining species richness, we conducted regressions for all possible combinations of the six climate variables and their quadratic terms and considered the model with the lowest value of small‐sample size corrected Akaike information criterion (AICc) to be the best model (Burnham & Anderson, 2002). We assessed the relative importance of each of the independent variables in each of the best regression models based on the absolute values of the standardized regression coefficients of the independent variables in the model.

**FIGURE 1:**
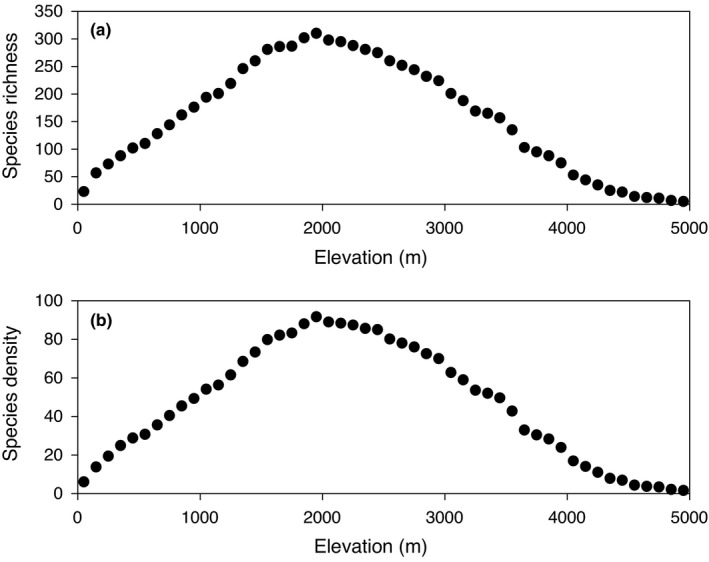
Relationships between elevation and species richness (a) or species density (b) for pteridophytes in Nepal. Each dot represents a 100‐m elevation band. Species density is defined as species richness in an elevational band being divided by the log_10_‐tranformed area (km^2^) of the elevational band

Surface area typically decreases with elevation (Körner, 2000), which might influence the species richness−climate relationship when the species−area relationship is not accounted for (Kessler, Hofmann, et al., 2011). To determine whether this is the case in our study, following previous studies (Guo et al., 2021; Qian, Deng, et al., 2019; Qian et al., 2021; Vetaas & Grytnes, 2002), we converted raw species richness per elevational band to species density by dividing the number of species in an elevation band by the log‐transformed area of the band. Because species density was nearly perfectly correlated with species richness (Pearson correlation coefficient = .997) for the 50 elevational bands (Figure 1), it is unlikely that variation in area between elevation bands has an effect on our results. We thus conducted all analyses using species richness.

Considering that pteridophytes do not form a monophyletic group (lycophytes are sister to the combination of ferns and seed plants; PPG I, 2016), in addition to analyzing the data for pteridophytes as a whole, we analyzed the data for lycophytes and ferns separately. This allows direct comparisons of the results of previous and future studies based on exclusively lycophytes or ferns with the results of the present study. Furthermore, previous studies (e.g., Bhattarai et al., 2004) have shown that species richness of pteridophytes increases with elevation up to 2000 m and then decreases with elevation. Accordingly, in addition to analyzing the data for the full elevational gradient (60−5000 m) as a whole, we also analyzed the data for the lower segment (60−2000 m) and the upper segment (2000−5000 m) of the elevational gradient separately.

We used SYSTAT (Wilkinson et al., 1992) and Spatial Analysis in Macroecology (www.ecoevol.ufg.br/sam/) for statistical analyses.

## 3 RESULTS

### 3.1 Spatial patterns of species richness and climatic variables

**FIGURE 2:**
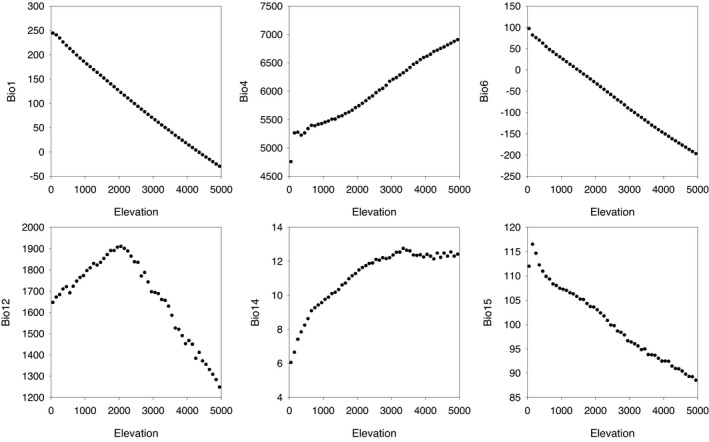
Relationships between elevation (m) and climatic variables along the elevational gradient in Nepal. The climatic variables are bio1 (mean annual temperature), bio4 (temperature seasonality), bio6 (minimum temperature of the coldest month), bio12 (annual precipitation), bio14 (precipitation of the driest month), and bio15 (precipitation seasonality). More information about the climatic variables is available at the website https://chelsa‐climate.org/bioclim/

Pteridophyte species richness increased from 23 species at 0−100 m to a maximum of 310 species at 1900−2000 m and then decreased to 5 species at 4900−5000 m (Figure 1). Annual mean temperature, temperature seasonality, minimum temperature of the coldest month, and precipitation seasonality decreased, in general, from low to high elevations (Figure 2). Annual precipitation increased from the elevational band at 0−100 m up to the elevational band at 2000−2100 m and then decreased upward (Figure 2). Precipitation seasonality increased from the elevational band at 0−100 m up to the elevational band at 3300−3400 m, from where it tended to level off (Figure 2).

### 3.2 Single‐variable analyses

**FIGURE 3:**
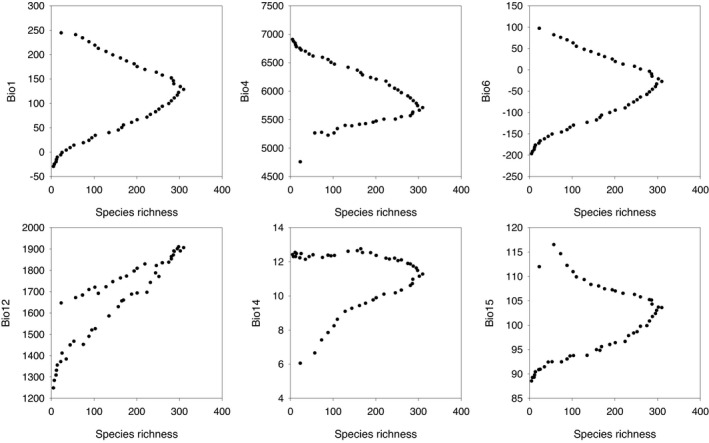
Relationships between pteridophyte species richness (SR) and climatic variables along the elevational gradient in Nepal. The climatic variables are bio1 (mean annual temperature), bio4 (temperature seasonality), bio6 (minimum temperature of the coldest month), bio12 (annual precipitation), bio14 (precipitation of the driest month), and bio15 (precipitation seasonality). More information about the climatic variables is available at the website https://chelsa‐climate.org/bioclim/. Note that we used y‐axes for climatic variables in order to make the panels in this figure directly comparable with those in Figure 2; we did not mean that variables on y‐axes represent dependent variables

The relationships between pteridophyte species richness and climatic variables tended to be hump‐shaped along the elevational gradient in the central Himalaya, except for annual precipitation (bio12), which was positively correlated with pteridophyte species richness (Figure 3). Across the full elevational gradient, the first‐order polynomial regression model with annual precipitation explained 82% of the variation in pteridophyte species richness, whereas the other five climatic variables explained only about 20% or less (Table 1). However, when the second‐order polynomial regression model was used, the amount of the variation in pteridophyte species richness explained by each of the climatic variables increased by more than four times in five of six cases (except for bio12) and ranged from 81.3% to 90.9%, with only bio14 remaining low at 30.9%. These results for pteridophytes as a whole were more similar to the results for ferns than the results for lycophytes (Table 1). When the 18 first‐order polynomial regression models were compared with the 18 second‐order polynomial regression models for pteridophytes, lycophytes, and ferns across the full elevational gradient, each second‐order polynomial model explained, on average, three times as much variation in species richness as did the first‐order polynomial model (77.5% vs. 25.2%). Regardless of which group of species was considered, species richness was only weakly associated with mean annual temperature (*R* ^2^_adj_ = .13−.20) but was strongly associated with annual precipitation (*R* ^2^_adj_ = .81−.82) (Table 1).

**TABLE 1:** Adjusted coefficients of determination (*R* ^2^_adj_) of the first‐order (1st) and second‐order (2nd) polynomial regressions of species richness of pteridophytes, lycophytes, and ferns on each of six climatic variables along the full, upper, and lower elevational gradients in the central Himalaya

Variable	Pteridophytes		Lycophytes		Ferns	
	1st	2nd	1st	2nd	1st	2nd
Full gradient						
bio1	0.133(+)	0.913	0.202(+)	0.874	0.127(+)	0.912
bio4	0.201(−)	0.813	0.272(−)	0.792	0.194(−)	0.811
bio6	0.145(+)	0.909	0.215(+)	0.868	0.139(+)	0.907
bio12	0.820(+)	0.860	0.821(+)	0.860	0.815(+)	0.854
bio14	0.003(+)	0.309	0.002(−)	0.436	0.004(+)	0.298
bio15	0.127(+)	0.847	0.193(+)	0.843	0.121(+)	0.843
Lower gradient						
bio1	0.991(−)	0.991	0.941(−)	0.964	0.990(−)	0.989
bio4	0.816(+)	0.945	0.825(+)	0.894	0.811(+)	0.945
bio6	0.992(−)	0.992	0.948(−)	0.964	0.991(−)	0.991
bio12	0.979(+)	0.979	0.926(+)	0.956	0.978(+)	0.977
bio14	0.936(+)	0.980	0.947(+)	0.955	0.930(+)	0.977
bio15	0.870(−)	0.951	0.868(−)	0.903	0.866(−)	0.950
Upper gradient						
bio1	0.983(+)	0.982	0.949(+)	0.949	0.981(+)	0.980
bio4	0.987(−)	0.987	0.947(−)	0.945	0.985(−)	0.986
bio6	0.984(+)	0.983	0.950(+)	0.949	0.982(+)	0.981
bio12	0.981(+)	0.983	0.919(+)	0.921	0.981(+)	0.983
bio14	0.365(−)	0.404	0.466(−)	0.473	0.356(−)	0.399
bio15	0.960(+)	0.967	0.937(+)	0.938	0.958(+)	0.965

Note

A positive or negative relationship in a first‐order polynomial regression was indicated with a plus or minus sign, respectively, in parentheses. Climatic variables: mean annual temperature (bio1), temperature seasonality (bio4), minimum temperature of the coldest month (bio6), annual precipitation (bio12), precipitation during the driest month (bio14), and precipitation seasonality (bio15).

When the lower and upper segments of the elevational gradient were considered separately, the amount of the variation in species richness explained by each of the six climatic variables did not differ greatly between the first‐order and the second‐order polynomial models, regardless of whether the lower or upper segment of the elevational gradient was considered and which group of species was considered (Table 1). For example, when averaging across the six climatic variables and the three groups of species (pteridophytes, lycophytes, ferns), the amount of the variation in species richness explained by each climatic variable was 92.3% and 96.1% with the first‐order and second‐order polynomial models, respectively, when the lower segment of the elevational gradient was considered, and 87.1% and 87.6% when the upper segment of the elevational gradient was considered. However, when the first‐order polynomial models for the lower segment of the elevational gradient were compared with their counterparts for the upper segment of the elevational gradient, the sign (i.e., positive or negative) of regression coefficient was opposite in 15 out of the 18 pairs of the models (Table 1). Of the six climatic variables, annual precipitation was the only variable having the consistent sign of regression coefficient between the two segments of the elevational gradient (Table 1).

### 3.3 Variance partitioning

**FIGURE 4:**
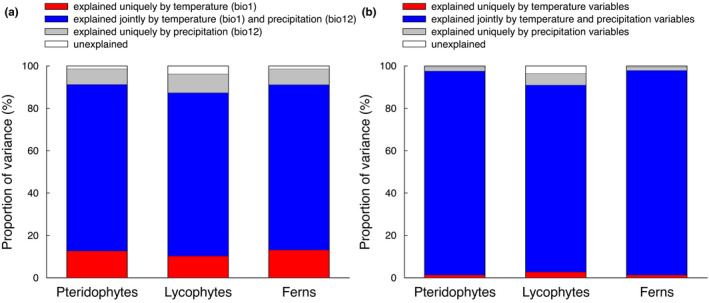
Variation in species richness of pteridophytes, lycophytes, and ferns explained only by temperature variables, only by precipitation variables, or jointly by temperature and precipitation variables for the elevational gradient in Nepal. The variation partitioning analyses presented in panel (a) included only the two main climate variables (mean annual temperature and annual precipitation); the variation partitioning analyses presented in panel (b) included six climatic variables, of which three were temperature variables (i.e., mean annual temperature, minimum temperature of the coldest month, temperature seasonality) and the other three were precipitation variables (i.e., annual precipitation, precipitation during the driest month, precipitation seasonality). Each variation partitioning analysis was based on second‐order polynomial regression

When species richness was simultaneously regressed on mean annual temperature and annual precipitation in the second‐order polynomial model, the two climatic variables explained 96.3%−98.7% of the variation in species richness for the three groups of species, with mean annual temperature uniquely explaining slightly more variation than annual precipitation for pteridophytes as a whole and for ferns (Figure 4a). However, the vast majority (87.4%−91.3%) of the variation in species richness for the three groups of species was explained jointly by mean annual temperature and annual precipitation (Figure 4a). When the second‐order polynomial models were expanded by including all the six climatic variables, the models explained nearly all the variation in species richness for each of the species groups, and the amount of the variation in species richness that was uniquely explained by either temperature‐related or precipitation‐related variables was reduced in each model (compare Figure 4a with 1b), even though precipitation‐related variables explained slightly more variation than temperature‐related variables (Figure 4b).

**FIGURE 5:**
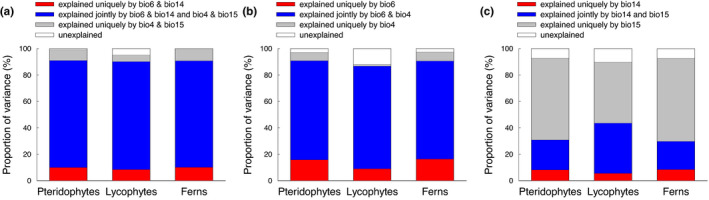
Variation in species richness of pteridophytes, lycophytes and ferns explained only by extreme climate variables, only by climate seasonality variables, or jointly by the extreme and seasonality climate variables for the elevational gradient in the central Himalaya. (a) Extreme climate variables included minimum temperature of the coldest month (bio6) and precipitation during the driest month (bio14), and climate seasonality variables included temperature seasonality (bio4) and precipitation seasonality (bio15). (b) Extreme climate variable was minimum temperature of the coldest month (bio6), and climate seasonality variables included temperature seasonality (bio4). (c) Extreme climate variables included precipitation during the driest month (bio14), and climate seasonality variables included precipitation seasonality (bio15). Each variation partitioning analysis was based on second‐order polynomial regression

When species richness was simultaneously regressed on the two variables representing extreme climate conditions (minimum temperature of the coldest month, precipitation during the driest month) and two variables representing climate seasonality (temperature seasonality, precipitation seasonality), the four climatic variables explained 95.2%−99.4% of the variation in species richness for the three groups of species, with 80.6%−81.7% of the variation being explained jointly by extreme climate and seasonality variables (Figure 5a). Extreme climate variables explained slightly more variation than climate seasonality for each of the three groups of species (Figure 5a). When the relative importance of extreme climate conditions and climate seasonality was examined separately for temperature and precipitation, the variation in species richness uniquely explained by minimum temperature of the coldest month was much greater than that uniquely explained by temperature seasonality for all the three groups of species (Figure 5b). However, the variation in species richness uniquely explained by precipitation during the driest month was much smaller than that uniquely explained by precipitation seasonality for all the three groups of species (Figure 5c).

**FIGURE 6:**
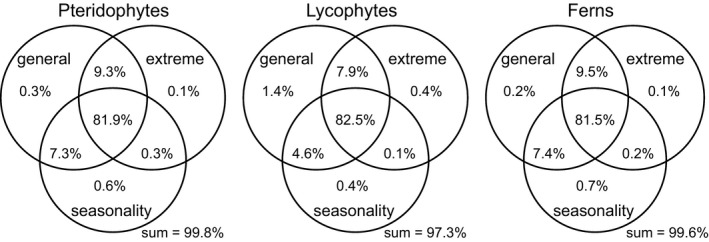
Pure and shared effects of three groups of climatic variables on species richness of pteridophytes, lycophytes, and ferns along the elevational gradient in the central Himalaya. The “general” group included mean annual temperature (bio1) and annual precipitation (bio12); the “extreme” group included minimum temperature of the coldest month (bio6) and precipitation during the driest month (bio14); the “seasonality” group included temperature seasonality (bio4) and precipitation seasonality (bio15). Each variation partitioning analysis was based on second‐order polynomial regression

When the variation in species richness explained by each full model was partitioned according to climate variables representing general conditions, climate variables representing extreme conditions, and climate variables representing seasonality, about 82% of the variation was explained jointly by the three types of climatic variables for each of the three species groups (Figure 6). For all the three species groups, the variation in species richness explained jointly by variables representing general and extreme climatic conditions (7.9%−9.5%) exceeded that explained jointly by variables representing general climatic conditions and seasonality (4.6%−7.4%), which in turn exceeded that explained jointly by variables representing extreme climatic conditions and seasonality (0.1%−0.3%) (Figure 6). The variation in species richness that was explained uniquely by each of the three groups of climatic variables was <1% except for general climate for lycophytes (Figure 6).

### 3.4 Climatic variables retained in the best models

When species richness of each of the three species groups (pteridophytes, lycophytes, ferns) for the full elevational gradient, or its lower or upper segment, was regressed on the six climatic variables in a polynomial model for all possible combinations of the 12 model terms (i.e., linear and quadratic terms), a total of 4095 models resulted, of which the one with the smallest AICc was considered the best model. Of the nine best models for the three species groups and three types of elevational gradients (full gradient, and its lower and upper segments), each model explained 96.5% or more variation in species richness and included 4 to 8 model terms (Table 2), with an average of 6.1. The total number of linear terms in the nine best models was nearly equal to that of quadratic terms (28 vs. 27; Table 2). The retained model terms were highly consistent between pteridophytes and ferns, which differed substantially from those for lycophytes, regardless of whether the full elevational gradient or its lower or upper segment was considered (Table 2).

**TABLE 2:** Adjusted coefficients of determination (*R* ^2^_adj_) and standardized coefficients from the best regression models of species richness of pteridophytes (P), lycophytes (L), and ferns (F) on six climatic variables along the full elevational gradient and the upper and lower segments of the elevational gradients in the central Himalaya

Variable	Full gradient			Lower segment			Upper segment		
	P	L	F	P	L	F	P	L	F
bio1	6.0		7.5				−1.3		−1.4
bio4	19.2		18.5	4.0			29.1	152.7	29.5
bio6	−11.1		−12.7	−1.8		−2.2		85.7	
bio12	−4.8	−9.1	−4.5		26.3		−3.1		−3.2
bio14		−0.4			−6.5	1.2			
bio15	−4.5		−5.8	0.3		1.1			
bio1 × bio1		−1.1					1.4	−18.6	1.4
bio4 × bio4	−25.2		−24.7	−4.6	0.3	−1.1	−30.7	−146.7	−31.0
bio6 × bio6		−2.2						60.7	
bio12 × bio12	4.3	8.2	4.2		−26.7	−0.3	2.9	−2.1	3.2
bio14 × bio14					7.5				
bio15 × bio15	4.4		5.6				−0.4		−0.5
*R* ^2^_adj_	.996	.965	.996	.997	.991	.997	.998	.983	.998

Note

Each best model was the one with the lowest AICc among the models of all possible combinations of the six climate variables and their quadratic terms. Climatic variables: mean annual temperature (bio1), temperature seasonality (bio4), minimum temperature of the coldest month (bio6), annual precipitation (bio12), precipitation during the driest month (bio14), and precipitation seasonality (bio15).

For the full elevational gradient, temperature seasonality (bio4) and its quadratic term were the two strongest factors, followed by minimum temperature of the coldest month (bio6), in the best models for pteridophytes as a whole and for ferns, based on the absolute values of standardized coefficients of the model terms (Table 2); annual precipitation (bio12) and its quadratic term were the most important factors for the best model of lycophytes (Table 2).

When the lower and upper segments of the elevational gradient were considered separately, temperature seasonality (bio4) and its quadratic term had the strongest effects for pteridophytes in both lower and upper segments of the elevational gradient and for ferns in the lower elevational segment (Table 2); minimum temperature of the coldest month had the strongest effect for ferns in the upper elevational segment (Table 2). The best models for lycophytes included annual precipitation and its quadratic term, followed by precipitation during the driest month (bio14) and its quadratic term, in the lower elevational segment whereas temperature seasonality and its quadratic term, followed by minimum temperature of the coldest month, had the strongest effects in the upper elevational segment. In other words, precipitation‐related variables were the main determinants of lycophytes in the lower elevational segment, whereas temperature‐related variables were the main determinants of lycophytes in the upper elevational segment (Table 2).

## 4 DISCUSSION

In this study, we used current data on the taxonomy and elevational distribution of pteridophytes in Nepal in combination with novel climate data to update and expand our understanding of the drivers of pteridophyte richness in the Himalaya, compared to the previous study of Bhattarai et al. (2004). The main results of our study can be summarized as showing that pteridophyte richness along the elevational gradient in Nepal is largely driven by a combination of both temperature‐ and precipitation‐related parameters, although precipitation‐related variables play a slightly stronger role, and that extreme low temperatures (at high elevations) and seasonal climate variability (at low elevations) are the strongest determinants of pteridophyte species richness.

The hump‐shaped richness pattern observed in this study for pteridophytes along the elevational gradient in Nepal is similar to that observed in Bhattarai et al. (2004). However, the number of species at the peak (at ~2000 m in elevation) in our study is about 1.5 times that observed in their study, whereas the number of species at either end of the gradient is similar in the two studies, so that the slope of the relationship between species richness and elevation on either side of the richness peak on the elevational gradient is much steeper in our study. Furthermore, the sources of climate data are different between the two studies. Whereas Bhattarai et al. (2004) used thirty local climate stations providing temperature lapse rate and interpolated precipitation, we used a global climate model based on remote sensing data (Karger et al., 2017). Accordingly and unsurprisingly, the relationships between species richness and climatic variables are substantially different between the two data sets in some cases. More generally, hump‐shaped elevational richness patterns have been found to be the rule on extensive tropical and subtropical elevational gradients (e.g., Hernández‐Rojas et al., 2020; Kessler, Kluge, et al., 2011; Khine et al., 2019; Salazar et al., 2015). Mostly, the authors of these studies interpreted this pattern as reflecting the physiological limitations of low temperatures at high elevations, and of high temperatures and lower precipitation at low elevations, leading to water stress under hot conditions. However, the relative roles of temperature‐ versus precipitation‐related variables and, in particular, the influence of climatic extremes and seasonality remain poorly explored.

In our study, we found that pteridophyte species richness was more strongly associated with annual precipitation than with mean annual temperature (Table 1). This suggests that precipitation plays a more important role than temperature in driving species richness of pteridophytes. Our results are consistent with those reported in previous studies for pteridophytes, not only along elevational gradients but also across regional scales. For example, the correlation between species richness and precipitation is stronger than that between species richness and temperature for pteridophyte assemblages across China, both at spatial scales of 226 km^2^ (Qian et al., 2012) and of ~144,000 km^2^ (Qian et al., 2021). Mean annual precipitation was also proposed to be the main correlate of pteridophyte species richness in the Iberian Peninsula (Ferrer‐Castan & Vetaas, 2005; Marquez et al., 1997), Bolivia (Kessler, 2001), Australia (Bickford & Laffan, 2006), and globally (Kreft et al., 2010; Weigand et al., 2020). This consistency among studies for different regions suggests that a stronger relationship of pteridophyte species richness with precipitation than with temperature is a general pattern, although the opposite pattern may appear in some regions (e.g., Bogonovich et al., 2014). The strong association of pteridophytes with water‐related variables may be because they have less active stomatal control (Brodribb & McAdam, 2011; McAdam & Brodribb, 2012), which leads to reduced water use efficiency (Kessler, Kluge, et al., 2011; Weigand et al., 2020).

On the other hand, our variation partitioning analyses showed that the majority of the variation in pteridophyte species richness was explained by temperature‐ and precipitation‐related variables jointly. This suggests that the hump‐shaped pattern of species richness along the elevational gradient is likely driven by the interplay of the two types of climatic variables. This interplay may influence species richness in several ways. First, the water−energy dynamic theory (WED; O'Brien, 1993) proposes that these two factors influence the available liquid water and thereby the biological activity of the ecosystem including the productivity and hence the chemical energy that is available for species to survive. The linear response in richness to precipitation and the parabolic response to thermal energy found by us are consistent with the WED (mean annual temperature is strongly correlated to potential evapotranspiration). In fact, Kessler et al. (2014) found that along an elevational gradient in Ecuador, pteridophyte richness is closely correlated to annual biomass increment of the ferns, indicating that energy availability may play a role in determining the upper number of pteridophyte species that can co‐occur. Alternatively, richness may be limited by the physiological tolerances of the individual species, so that different parameters may be limiting under different conditions, implying that a combination of factors represents the best model. For example, along an elevational gradient, the upper distributional limits of species may be determined by their individual tolerance to low temperatures or frost, whereas the lower limits may be determined either directly by the tolerance to high temperatures or by the water stress induced by high temperatures. For example, Kessler (2001) found that Andean fern species can extend their distributions into the Amazon lowlands where precipitation is high year‐round.

Distinguishing between the two underlying causes determining a relationship between pteridophyte richness and combined temperature–precipitation variables is not trivial. There is no explicit interaction term in the original WED model (O'Brien, 1993), although Vetaas et al. (2019) introduced an interaction between the length of growing season and precipitation in a seasonal WED model that explained 98% of the variation in pteridophyte richness in Nepal, even though the precipitation term as such was insignificant. Likewise, in a study including eight elevational gradients of pteridophyte diversity across East Asia, Khine et al. (2019) found that actual evapotranspiration, which also combines temperature‐ and precipitation‐related parameters, is a powerful predictor of local pteridophyte richness. Yet, while these studies further emphasize the importance of considering interactions between temperature‐ and precipitation‐related variables, they do not allow disentangling the underlying causes.

One potential approach is to focus on the importance of climatic extremes, since they are likely to be the actual physiologically limiting factors leading to mortality among species, whereas ecosystem productivity is more likely to be driven by the temporally most common factors (Körner & Hiltbrunner, 2018). We found that winter low temperature was a stronger determinant of pteridophyte species richness than either annual mean temperature or temperature seasonality. This is consistent with the findings of several previous studies. For example, in China, species richness of amphibians (Qian et al., 2007), seed plants (Qian, Deng, et al., 2019; Wang et al., 2011), and pteridophytes (Qian et al., 2021) is strongly associated with winter temperature. In addition, many studies on phylogenetic structure, including those for elevational gradients (e.g., Hernández‐Rojas et al., 2021), have also found that minimum temperature (commonly measured as minimum temperature of the coldest month) is more strongly associated with phylogenetic metrics than is temperature seasonality. Taken together, it appears that minimum temperature is commonly a stronger determinant of species richness and community composition than either annual mean temperature or temperature seasonality. This in turn indicates that it is the physiological tolerance to extreme low temperatures that determines the upper latitudinal and elevational limits of many individual species and hence limits species richness. For example, among ectotherm animals, there is a close correlation between tolerance to low temperatures and latitudinal distribution (Sunday et al., 2014). Interestingly, no such correlation was found by Sunday et al. (2014) for heat tolerance and the distribution towards low latitudes, corroborating that upper and lower distributional limits are determined by different sets of factors.

Indeed, when comparing the effects of climate dryness and precipitation seasonality on pteridophyte species richness, we found that the latter is more strongly associated with pteridophyte species richness than is the former. This pattern is contrary to that for the effects of extreme temperatures as discussed above and indicates that it is the duration at which pteridophytes are under water stress that limits pteridophyte distributions. This makes intuitive sense, since low temperatures can vary enormously and reach lethal levels, whereas low precipitation cannot decrease below “no precipitation,” so that it is the length of the dry spell that is limiting for plants. Generally, it is well known that pteridophytes proliferate in humid environments, especially in cloud forests where air humidity is consistently high (Karger et al., 2021; Kessler, Kluge, et al., 2011; Khine et al., 2019). In tropical regions such as Uganda and western Amazonia, soil moisture has also been found to play a significant role in determining pteridophyte richness (Lwanga et al., 1998; Tuomisto & Poulsen, 1996). Many authors have proposed that the importance of climatic dryness for pteridophytes is related to the dependence on external water for reproduction (Lehmann et al., 2002; Pausas & Sáez, 2000). However, as previously mentioned, it may also be linked to the poor ability of pteridophytes to control transpiratory water loss (Brodribb & McAdam, 2011; McAdam & Brodribb, 2012). Here, ecophysiological studies are needed to better understand the role of water limitation on pteridophyte growth and reproduction.

In conclusion, our study suggests that pteridophyte richness in the Nepalese Himalaya is limited by low winter temperatures at high elevations and by the length of dry periods at low elevations, with maximum richness under conditions of moderate temperatures and constantly high humidity as found in cloud forests at mid‐elevations. The robustness of these conclusions should be tested along other elevational gradients, because minimum temperature is, in general, less strongly correlated with temperature seasonality along elevational gradients (Janzen, 1967), compared to the latitudinal gradient. If confirmed, our study shows that reactions of pteridophyte species to climate change may differ along the upper and lower limits of their elevational ranges, since shifts in dry season length and winter temperatures may differ in direction, magnitude, and extent.

## AUTHOR CONTRIBUTIONS

**Hong Qian:** Conceptualization (lead); Data curation (lead); Formal analysis (lead); Investigation (lead); Writing – original draft (lead); Writing – review & editing (lead). **Michael Kessler:** Writing – original draft (equal); Writing – review & editing (equal). **Ole Reidar Vetaas:** Writing – original draft (supporting); Writing – review & editing (equal).

## CONFLICT OF INTEREST

The authors declare no conflict of interest.

## Supporting information

Table S1

## Data Availability

All data used in this study have been published and are accessible to readers from the cited sources. The data on which the analyses of this study were based are available at https://github.com/Kifir0411/ECE‐2021‐12‐01997.
