# Supplementary material for: Pteridophyte species richness in the central Himalaya is limited by cold climate extremes at high elevations and rainfall seasonality at low elevations
Source: Ecol Evol. 2022 May 24;12(5):e8958. doi: 10.1002/ece3.8958 (PMC9130305; doi:10.1002/ece3.8958)
Supplement: Supplementary file 1 — Table S1 [file ECE3-12-e8958-s001.doc]

**Supporting Information**

Table S1. Number of genera and species in each family of pteridophytes in Nepal.

| Class | Family | Numb. of genera | Numb. of species |
| --- | --- | --- | --- |
| Lycopodiopsida | Isoetaceae | 1 | 1 |
| Lycopodiopsida | Lycopodiaceae | 6 | 14 |
| Lycopodiopsida | Selaginellaceae | 1 | 23 |
| Polypodiopsida | Aspleniaceae | 2 | 34 |
| Polypodiopsida | Athyriaceae | 5 | 58 |
| Polypodiopsida | Blechnaceae | 3 | 3 |
| Polypodiopsida | Cyatheaceae | 2 | 6 |
| Polypodiopsida | Cystopteridaceae | 3 | 7 |
| Polypodiopsida | Davalliaceae | 1 | 7 |
| Polypodiopsida | Dennstaedtiaceae | 5 | 15 |
| Polypodiopsida | Diplaziopsidaceae | 1 | 1 |
| Polypodiopsida | Dipteridaceae | 1 | 1 |
| Polypodiopsida | Dryopteridaceae | 7 | 106 |
| Polypodiopsida | Equisetaceae | 1 | 2 |
| Polypodiopsida | Gleicheniaceae | 2 | 5 |
| Polypodiopsida | Hymenophyllaceae | 3 | 12 |
| Polypodiopsida | Hypodematiaceae | 2 | 2 |
| Polypodiopsida | Lindsaeaceae | 3 | 4 |
| Polypodiopsida | Lygodiaceae | 1 | 4 |
| Polypodiopsida | Marattiaceae | 1 | 2 |
| Polypodiopsida | Marsileaceae | 1 | 1 |
| Polypodiopsida | Nephrolepidaceae | 1 | 4 |
| Polypodiopsida | Oleandraceae | 1 | 3 |
| Polypodiopsida | Onocleaceae | 1 | 1 |
| Polypodiopsida | Ophioglossaceae | 6 | 11 |
| Polypodiopsida | Osmundaceae | 2 | 2 |
| Polypodiopsida | Plagiogyriaceae | 1 | 2 |
| Polypodiopsida | Polypodiaceae | 14 | 67 |
| Polypodiopsida | Psilotaceae | 1 | 1 |
| Polypodiopsida | Pteridaceae | 18 | 84 |
| Polypodiopsida | Salviniaceae | 2 | 2 |
| Polypodiopsida | Tectariaceae | 1 | 5 |
| Polypodiopsida | Thelypteridaceae | 15 | 38 |
| Polypodiopsida | Woodsiaceae | 2 | 6 |
